# Assembly of lipid droplet-associated ring structures in hepatitis C virus-infected cells via liquid–liquid phase separation (LLPS) and non-LLPS mechanisms

**DOI:** 10.1128/jvi.00780-26

**Published:** 2026-06-26

**Authors:** Mengyu Jiao, Alu Konno, Xiaowei Wang, Jie Liu, Masahiko Ito, Shinya Satoh, Ryosuke Suzuki, Yasumasa Iwatani, Tetsuro Suzuki

**Affiliations:** 1 Department of Microbiology and Immunology, Hamamatsu University School of Medicine, Shizuoka, Japan; 2 Department of Virology II, National Institute of Infectious Disease, Japan Institute for Health Security, Tokyo, Japan; 3 Next Generation Creative Education Center for Medicine, Engineering, and Informatics (Nx-CEC), Hamamatsu University School of Medicine, Shizuoka, Japan; Wake Forest University School of Medicine, Winston-Salem, North Carolina, USA

**Keywords:** hepatitis C virus, liquid-liquid phase separation, stress granule

## Abstract

Hepatitis C virus (HCV) exploits lipid droplets (LDs) during the production of infectious particles. Although several host factors, including stress granule (SG) proteins, have been reported to localize to LDs in HCV-infected cells, the mechanism by which these proteins are recruited to LDs has remained unclear. In this study, we show that Ras-GAP SH3 domain-binding protein 1 (G3BP1), an essential SG component, is recruited to LDs with a time lag of several hours or more after the HCV Core protein accumulates on LDs following infection, and that G3BP1 retains its ability to assemble SGs even when localized to LDs during the late phase of infection. Treatment of HCV-infected cells with compounds that disrupt liquid–liquid phase separation (LLPS) abolished the LD localization of G3BP1, as well as another SG protein, T-cell intracellular antigen-1, whereas the LD localization of the viral proteins Core and NS5A was not affected. Finally, we demonstrate that ectopic expression of HCV Core alone is insufficient to recruit G3BP1 to LDs; however, co-expression of Core together with subgenomic HCV RNA, even a replication-defective replicon RNA, leads to the accumulation of G3BP1 around LDs. Collectively, these findings suggest that during the HCV life cycle, the initial localization of Core to LDs, followed by the recruitment of newly synthesized viral RNA to the LD surface, drives LLPS-mediated assembly of biomolecular condensates containing G3BP1 and other host SG proteins around LDs.

**IMPORTANCE:**

During the late stages of hepatitis C virus (HCV) infection, various viral and host proteins are known to accumulate around lipid droplets (LDs), where virion assembly is thought to occur. However, it has remained unclear how the expression of a limited subset of viral factors can induce such an extensive cellular reorganization. Here, we identify that HCV infection induces the relocalization of the stress granule (SG)-associated proteins G3BP1 and TIA-1 onto LDs through liquid–liquid phase separation (LLPS). We further demonstrate that the minimal requirement for G3BP1 recruitment to LDs is the coexistence of the HCV Core and the viral RNA. Because G3BP1 plays a central role in the interaction network underlying SG formation and is capable of mobilizing additional SG components, our findings suggest that LLPS-dependent assembly of biomolecular condensates occurs around LDs during HCV infection. This work provides mechanistic insight into HCV particle formation, including viral genome packaging, and offers a conceptual basis for developing future antiviral strategies.

## INTRODUCTION

Hepatitis C virus (HCV), a positive-sense single-stranded RNA virus of the genus Hepacivirus, establishes replication in hepatocytes through a tightly orchestrated interplay with host lipid and membrane metabolisms (1). After receptor-mediated entry and uncoating, the viral RNA is translated at the endoplasmic reticulum (ER) to generate a polyprotein that is cleaved into structural and nonstructural (NS) proteins, the latter reorganizing ER-derived membranes into replication organelles (2, 3). A hallmark of the HCV life cycle is its exploitation of cytosolic lipid droplets (LDs) (4): capsid protein Core and viral NS proteins, such as NS5A, are targeted to LD surfaces, where they coordinate the assembly of replication complexes and the formation of nascent virions (5). This coupling of RNA replication to LD-associated particle morphogenesis distinguishes HCV from other members of the Flaviviridae.

Eukaryotic cells are compartmentalized by various organelles for the temporal and spatial regulation of biochemical reactions. Some of them, such as mitochondria or lysosomes, maintain their independence by limiting their membranes. On the other hand, weak, multivalent interaction networks can assemble organelles without limiting membrane (also known as membrane-less organelles or biomolecular condensates) through the process called liquid–liquid phase separation (LLPS) (6, 7). Stress granules (SGs), assembled in the cells exposed to various stresses, including virus infection, are one of the most extensively studied biomolecular condensates (8–10). The central node of the LLPS molecular interactions for SG assembly is Ras-GAP SH3-binding protein 1 (G3BP1) (11–13). As seen in a variety of positive-strand RNA viruses, HCV infection imposes substantial stress on host cells, triggering antiviral stress responses (14). These virus-induced perturbations reflect a dynamic interplay between cellular attempts to restrict infection and viral strategies that counteract or exploit stress signaling to ensure productive replication. In general, SGs are assembled as a result of global translational shutdown in the virus-infected cells, and their potential antiviral roles are suggested (15). Indeed, many viruses have evolved mechanisms targeting G3BP1 to counteract stress granule assembly (reviewed in Brownsword and Locker [10]; see also Discussion). Orthoflaviviruses, including Zika virus (16–18), dengue virus (17), Japanese encephalitis virus (19), and West Nile virus (20), have been reported to suppress the SG assembly during infection. Similar to many other viruses, HCV induces SG assembly at the early phase of infection (21). At later stages of infection, however, an early report concluded that HCV inhibits SG formation (21), while others showed that SG-forming capacity is retained in infected cells (22, 23). Although HCV is also known to recruit G3BP1, along with various host proteins, onto LDs (21), the mechanism of the recruitment was poorly understood. Given the fact that G3BP1 is a multivalent interactor playing a central role in the LLPS network for SG assembly, we hypothesized that HCV recruits G3BP1 onto LDs via LLPS.

In this study, we show that G3BP1 forms two distinct structures—SGs and ring-like assemblies around LDs—which can coexist in the same cell. Unlike other viruses targeting G3BP1, HCV-infected cells retained the ability to assemble SGs even under arsenite-induced stress. Time-lapse immunofluorescence revealed that the HCV Core first localizes to LDs, followed by delayed recruitment of G3BP1. Using LLPS-modulating drugs, we found that G3BP1 and TIA-1 are recruited to LDs via LLPS, whereas the viral Core and NS5A localization remained unchanged. Coexpression experiments showed that recruitment of G3BP1 requires both Core and viral RNA. These findings suggest that newly synthesized HCV RNA associates with Core on LDs, thereby attracting G3BP1 through LLPS. This reveals a novel LLPS-based mechanism by which HCV hijacks SG proteins and provides insight into the late stages of the viral life cycle.

## RESULTS

### SG formation during HCV JFH-1 infection

**Fig 1:**
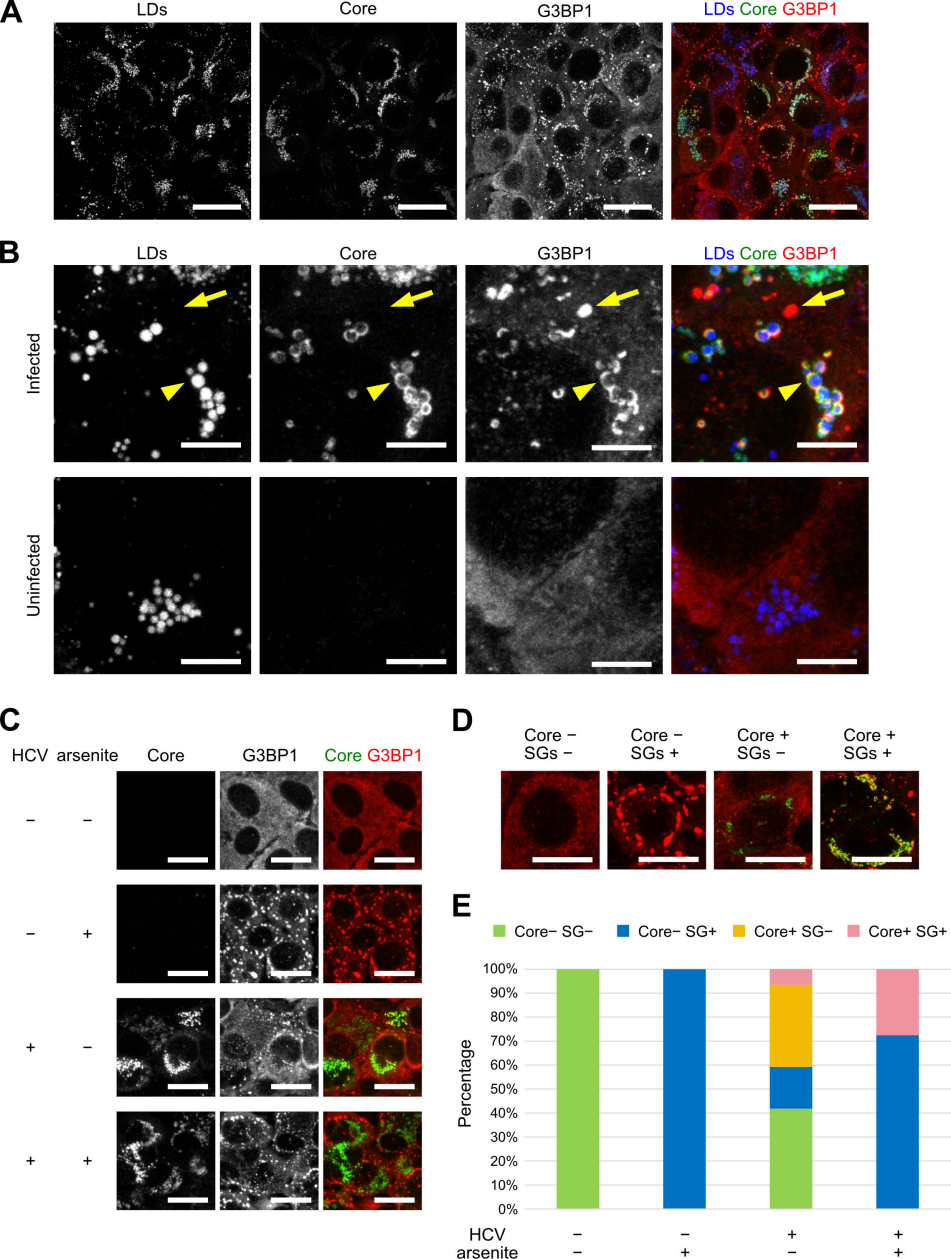
G3BP1 accumulates in two distinct types of compartments in HCV-infected cells. (**A**) Immunofluorescence confocal microscopy of HCV-infected Huh-7.5.1 cells at 48 hpi. Cells were stained with anti-HCV Core (green) and anti-G3BP1 (red) antibodies, and LDs with Lipi-Blue (blue). Scale bar, 20 µm. (**B**) Enlarged images showing two distinct G3BP1-enriched structures in the infected cell (upper panels). The yellow arrows indicate an SG devoid of LDs and Core protein. The yellow arrowheads indicate a ring-like structure surrounding LDs, where G3BP1 colocalizes with Core. G3BP1 shows diffuse localization in uninfected cells (lower panels). Scale bar, 5 µm. (**C**) Immunofluorescence confocal microscopy of HCV-infected or non-infected cells treated with sodium arsenite (arsenite) or untreated. Scale bar, 20 µm. (**D**) Intracellular distribution patterns of SG and Core. SG was evaluated based on G3BP1 localization detection. Scale bar, 10 µm. (**E**) The pattern defined in panel D was quantified in HCV-infected and non-infected cells, with or without arsenite treatment. Note that the absence of a detectable Core signal does not necessarily indicate the absence of infection. In total, 1,800 cells were counted. The counts for each group were as follows: 287 were HCV-negative and arsenite-negative (HCV‒, arsenite‒), 322 were HCV-negative and arsenite-positive (HCV‒, arsenite+), 471 were HCV-positive and arsenite-negative (HCV+, arsenite‒), and 720 were HCV-positive and arsenite-positive (HCV+, arsenite+). All experiments from panels A to C were performed twice independently, yielding similar results. Each image shows the maximum projection of confocal stacks; the graph in panel E is from one of the two independent experiments with similar results.

As described above, the relationship between HCV infection and SG formation has not been consistently demonstrated, and seemingly contradictory results have been reported (21, 22). To determine whether HCV infection can induce SG formation, we first infected Huh-7.5.1 cells with the HCV J6/JFH1 strain at a multiplicity of infection (MOI) of 1 for 2 h (hereafter referred to as “HCV-infected cells” under this condition). At 48 h post-infection (hpi), the localizations of G3BP1 and the HCV Core were examined by immunofluorescence confocal microscopy. At this time point, HCV-infected cells showed heterogeneous patterns of G3BP1 and HCV Core localization (Fig. 1A). In most HCV Core-positive cells, the Core localized around spherical LDs and appeared as ring-like structures in single confocal optical sections, consistent with previous reports (21, 24–26). We hereafter refer to this ring-like distribution of HCV Core surrounding LDs as a “peri-LD ring.” Note that this is actually a continuous thin layer partially or completely covering the surface of LDs.

Under culture conditions without HCV infection, G3BP1 is diffusely distributed throughout the cytoplasm. In HCV Core-positive cells, G3BP1 displayed either a diffuse cytoplasmic pattern or two distinct types of cytoplasmic foci. One type corresponded to the peri-LD ring, where G3BP1 colocalized with the HCV Core and appeared as partial or complete rings in confocal optical sections (Fig. 1B, arrowheads). Notably, peri-LD rings composed solely of G3BP1 without HCV Core were not observed. The other type of focus consisted of dot-like structures corresponding to SGs, which did not colocalize with HCV Core and did not contain LDs (Fig. 1B, arrows). Consistent with previous reports, SGs were observed only in a small fraction of cells at 48 hpi (Fig. 1B, C, and E) (22, 27, 28). Sodium arsenite treatment, a potent oxidative stress inducer (29), triggered robust SG formation in nearly all cells regardless of HCV infection (Fig. 1C, D, and E). These results indicate that at least a subset of HCV-infected cells can assemble SGs even without arsenite treatment, and that HCV infection does not impair the cellular capacity to form SGs.

### Recruitment of G3BP1 to lipid droplets follows the localization of HCV Core protein in infected cells

**Fig 2:**
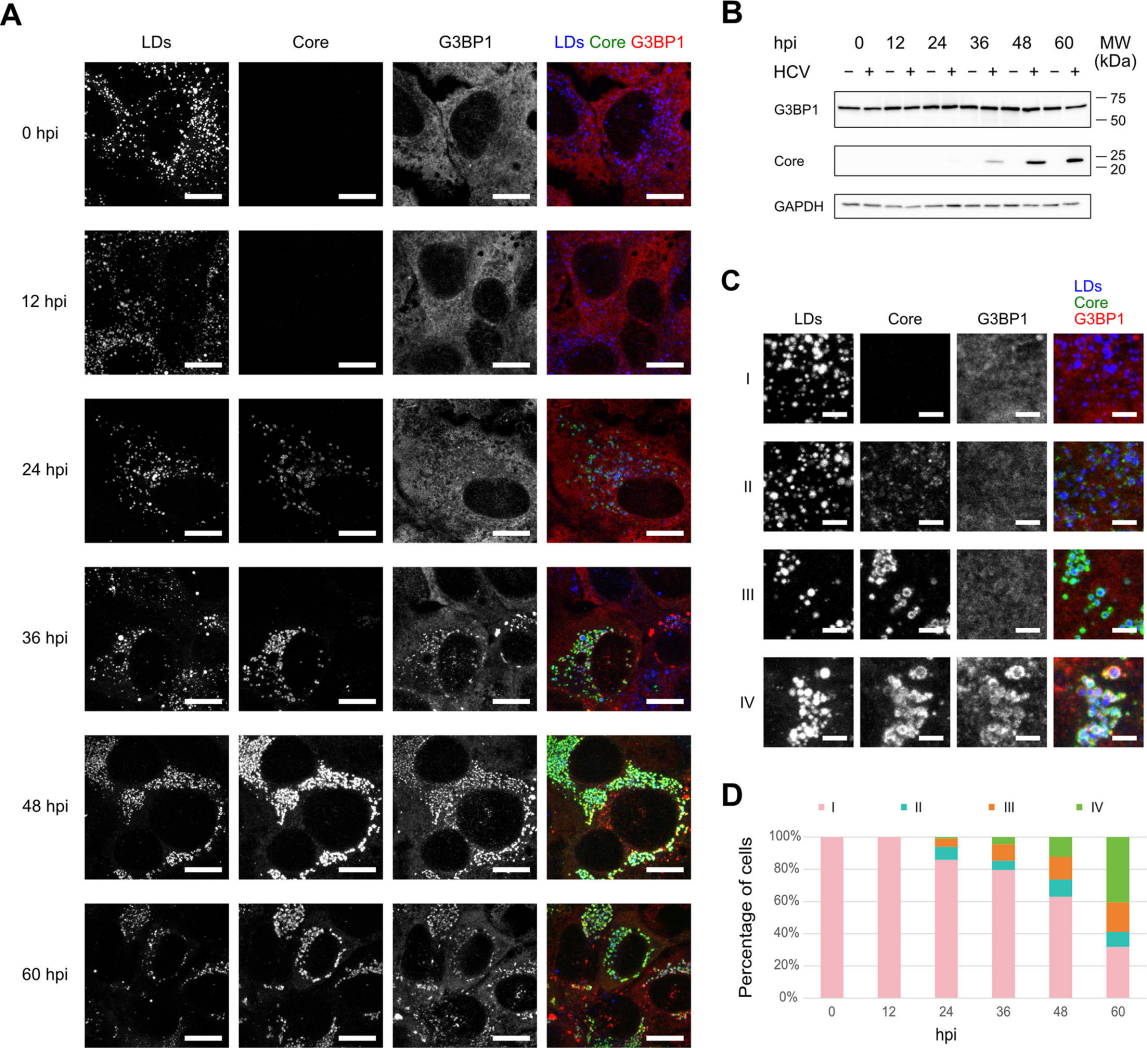
A temporal delay exists between the localization of Core protein on peri-LD rings and G3BP1 recruitment. (**A**) Time-dependent changes of G3BP1 and Core in Huh-7.5.1 cells infected with HCV (MOI = 1) from just before infection (0 hpi) to 12 to 60 hpi. Representative patterns from the acquired images are shown. Scale bar, 10 µm. (**B**) Western blotting of G3BP1 and Core expression at the indicated time points after infection. GAPDH was used as the loading control. (**C**) Classification of major localization patterns during infection: Class I, Core-negative/G3BP1 diffuse; Class II, granular Core/G3BP1 diffuse; Class III, peri-LD Core rings/G3BP1 diffuse; Class IV, peri-LD colocalization of Core and G3BP1. Scale bar, 2 µm. (**D**) Quantification of the proportion of each class as above at the indicated time points. All experiments were performed twice independently, yielding similar results. In total, 3,320 cells were counted. The counts for each group were 255 (0 hpi), 339 (12 hpi), 724 (24 hpi), 805 (36 hpi), 745 (48 hpi), and 452 (60 hpi). Each image shows the maximum projection of confocal stacks; the graph in panel D is from one of the two independent experiments with similar results.

As shown in Fig. 1A, at 48 hpi, the intracellular localization of HCV proteins and G3BP1 varied among cells. To elucidate the temporal order and intracellular dynamics of G3BP1 and HCV Core localization, Huh-7.5.1 cells infected with the HCV J6/JFH1 strain were fixed every 12 h and analyzed by confocal microscopy for the localization of LDs, G3BP1, and HCV Core (Fig. 2A). Because dead cells became apparent at 72 hpi, 60 hpi—when no obvious morphological alterations in cellular organelles were observed—was chosen as the endpoint for analysis.

HCV Core signals first appeared as cytoplasmic puncta at 24 hpi. At this time point, a small fraction of cells exhibited a peri-LD ring pattern of the Core, which became more prominent at 36 hpi as the signal intensity increased. Until 24 hpi, G3BP1 showed a diffuse and nearly uniform cytoplasmic distribution; however, from 36 hpi onward, in a subset of cells where HCV Core formed rings, G3BP1 colocalized with the Core within the peri-LD rings. Throughout the observation period, peri-LD rings consisting solely of G3BP1 without HCV Core were not detected. Even in cells displaying strong peri-LD ring localization of G3BP1, diffuse cytoplasmic G3BP1 staining remained detectable. Western blot analysis revealed that HCV Core appeared as a faint band at 24 hpi and increased in expression up to 60 hpi. In contrast, the total G3BP1 protein level remained nearly constant over time, regardless of HCV infection (Fig. 2B).

Based on these observations, the localization patterns of HCV Core and G3BP1 around LDs in infected cells were categorized into four classes (Fig. 2C). These were defined as follows: (Class I) no detectable HCV Core signal, with diffusely distributed cytoplasmic G3BP1; (Class II) punctate cytoplasmic HCV Core signals with diffusely distributed G3BP1; (Class III) peri-LD ring localization of the Core with diffusely distributed cytoplasmic G3BP1; and (Class IV) colocalization of the Core protein and G3BP1 within peri-LD rings. Although punctate and peri-LD ring signals frequently coexisted within the same cell, cells were classified as peri-LD ring-positive when at least one complete ring structure was detected. For G3BP1, cells were assigned to Class IV whenever G3BP1 colocalized with HCV Core around LDs, regardless of whether the ring pattern was partial or complete. The time-dependent changes in the distribution of these classes are shown in Fig. 2D. At 24 hpi, Class II cells—those exhibiting punctate HCV Core signals—were relatively abundant, whereas the proportion of Class III cells, containing peri-LD rings of HCV Core alone, gradually increased over time. The proportion of Class IV cells was lowest at 24 hpi but reached the highest among all classes at 60 hpi (Fig. 2D). These observations suggest that HCV Core protein first appears as punctate structures, subsequently forms peri-LD rings surrounding LDs, and finally recruits G3BP1 onto these peri-LD rings after a delay, likely of several hours, following the establishment of HCV Core-mediated LD rings.

### G3BP1 is recruited to LDs via LLPS-mediated interactions

**Fig 3:**
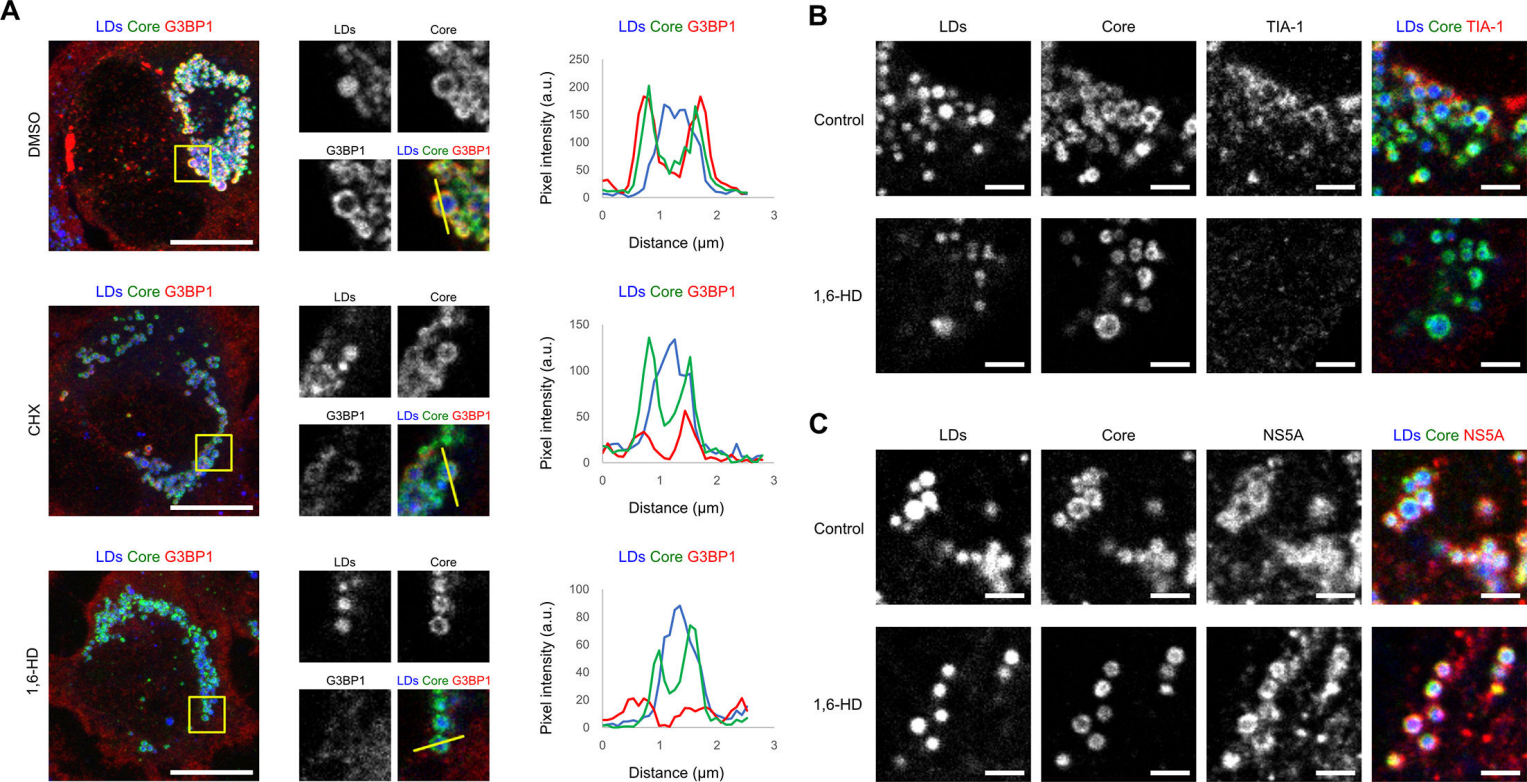
Drugs that disrupt LLPS abolish G3BP1 localization on peri-LD rings but do not affect Core localization. (**A**) HCV-infected Huh-7.5.1 cells (MOI = 1) were treated with cycloheximide (CHX), 1,6-hexanediol (1,6-HD), or DMSO (vehicle control for CHX). Left panels: lower-magnification confocal images stained with anti-Core (green), anti-G3BP1 (red), and Lipi-Blue (LDs, blue). Maximum-projection images are indicated. Scale bar, 10 µm. Middle panel sets: magnified views of boxed regions in left panels. Single optical sections are shown. Lines indicate where fluorescence intensity line profiles were obtained. Right panels: line-scan profiles of the three fluorescence channels. Brightness adjustments were applied to the middle panels for clarity, but line profiles were generated from raw images. (**B, C**) Effect of 1,6-HD on the peri-LD localization of the SG protein TIA-1 (**B**) and HCV NS5A (**C**). Scale bar, 2 µm. All experiments were performed twice independently, yielding similar results. Left images in panel A are the maximum projection of confocal stacks, and other images are single optical sections.

G3BP1 is a central node of the LLPS network that drives SG formation through weak multivalent interactions (11–13). It has been reported that G3BP1 binds the 5′ UTR of HCV RNA and plays a role in viral replication (30). We also found that HCV production is suppressed by knockdown of G3BP1. Specifically, the HCV Core protein level was reduced in persistently infected Huh-7 cells (HC-PI cells) (31) into which G3BP1 siRNA had been introduced, and intracellular HCV RNAs were also suppressed after inoculating naive Huh7.5.1 cells with the culture supernatant of these HC-PI cells (Fig. S1A and B). A previous immunoprecipitation study failed to detect an interaction between G3BP1 and the HCV Core protein (22), suggesting that any potential interaction between them is likely to be weak. In general, LLPS is driven by transient and low-affinity interactions, which are often lost during the stringent washing steps involved in affinity purification techniques, such as immunoprecipitation (32). Based on these observations, we hypothesized that the HCV Core protein recruits G3BP1 to the vicinity of LDs through LLPS-mediated interactions. To test this hypothesis, HCV-infected cells were treated with the established LLPS inhibitors 1,6-hexanediol (1,6-HD) (33) and cycloheximide (CHX) (34, 35) at 48 hpi, and the localizations of HCV Core and G3BP1 were analyzed by immunofluorescence microscopy. In untreated cells at 48 hpi, confocal images and line profile analyses revealed marked colocalization of HCV Core and G3BP1 within peri-LD ring structures (Fig. 3A). CHX treatment, which reduces the availability of free mRNAs by inhibiting their release from polysomes and thereby indirectly suppresses mRNA-mediated LLPS required for SG formation, partially decreased the level of colocalization between G3BP1 and HCV Core aroundd LDs (Fig. 3A). In contrast, 1,6-HD treatment completely abolished the peri-LD ring localization of G3BP1, likely due to disruption of hydrophobic interactions essential for LLPS. Neither treatment noticeably affected the localization of the HCV Core itself (Fig. 3A).

To quantify these observations, colocalization analysis was performed using Pearson’s correlation coefficient (PCC), which ranges from 1 (perfect colocalization) to –1 (complete exclusion), with 0 indicating random distribution. The PCC values for HCV Core protein and G3BP1 in DMSO-, CHX-, and 1,6-HD-treated cells were 0.68 ± 0.09, 0.46 ± 0.08, and 0.05 ± 0.16 (mean ± standard deviation, 100 × 100-pixel areas from seven different cells for each group), respectively, supporting the above observations. Western blot analysis showed that the levels of HCV Core, G3BP1, and GAPDH remained nearly unchanged before and after drug treatment (Fig. S1C), indicating that the loss of G3BP1 localization was not due to protein degradation. When cells were treated with 1,6-HD at 12 hpi, the staining pattern of G3BP1 was similar to that of untreated cells (Fig. S1C), and since Core was not detected at this point, virtually all cells remained in Class I. This suggests that no LLPS-mediated structures were formed in Class I cells.

We further examined the effects of 1,6-HD and CHX on the localization of the host SG protein TIA-1 and the viral protein NS5A. TIA-1 is an SG marker known to be recruited around LDs during HCV infection (28), whereas NS5A is a component of the viral replication complex localized to the ER and LDs (24). Treatment with 1,6-HD completely disrupted the peri-LD ring localization of TIA-1 (Fig. 3B and Fig. S2), whereas the ER and LD localization of NS5A was unaffected (Fig. 3C and Fig. S3). Together, these results indicate that the peri-LD ring localization of the SG proteins G3BP1 and TIA-1 is mediated by LLPS, whereas the localization of the viral proteins, HCV Core and NS5A, is not.

Next, the effect of 1,6-HD on HCV production was evaluated. Because 1,6-HD exhibits high cytotoxicity, LLPS studies typically involve treatment at a 5% concentration for 5 min. In the experiment shown in Fig. 3A, treatment under these conditions, followed immediately by analysis, revealed a change in the localization of G3BP1 around the LD. On the other hand, after removing the drug following the 5-min treatment and continuing culture for 1 h, G3BP1 was shown to redistribute around the LD (Fig. S4A). This indicates that the effect of 1,6-HD is reversible under the above conditions. After such 5-min 1,6-HD treatment, the culture medium was changed to 1,6-HD-free and cultured for 48 h, after which intracellular HCV RNA was quantitatively measured. No significant difference in intracellular viral RNA levels was observed between the 1,6-HD-treated group and the untreated group (Fig. S4B).

### Recruitment of G3BP1 to LDs requires the coexistence of HCV Core protein and viral RNA

**Fig 4:**
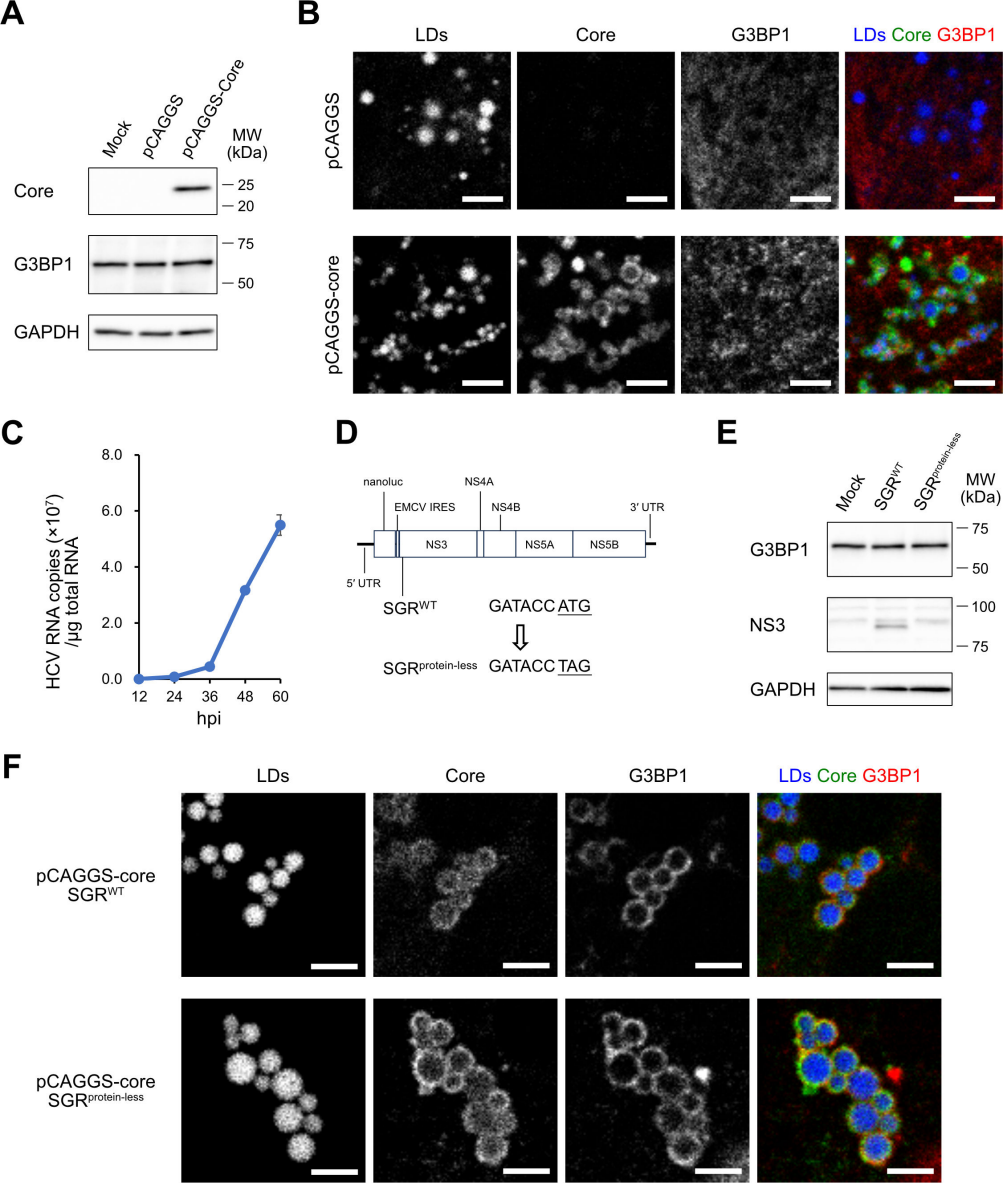
HCV Core protein and subgenomic replicon (SGR) RNA are the minimal requirements for G3BP1 recruitment around lipid droplets. (**A**) Detection of Core protein in Huh-7.5.1 cells transfected with pCAGGS-Core or pCAGGS (empty vector) by western blotting. (**B**) Immunofluorescence microscopy of Core and G3BP1 in the transfected cells as above. Scale bar, 2 µm. (**C**) Time course of HCV RNA levels in the virus-infected cells. The viral RNA copies in the cells at the indicated time points after infection were determined. (**D**) Schematic diagrams of wild-type (SGR^WT^) and protein-null mutant (SGR^protein-less^) subgenomic replicons. In SGR^protein-less^ subgenomic replicon construct, the start codon of the polyprotein is changed to a stop codon (under bars). (**E**) Western blotting of G3BP1 and HCV NS3 in cells transfected with SGR^WT^ or SGR^protein-less^ RNA. (**F**) Subcellular localization of Core and G3BP1 in cells transfected with Core-expression plasmid (pCAGGS-Core) together with SGR^WT^ RNA or SGR^protein-less^ RNA. All experiments were performed twice independently, yielding similar results. All confocal images are single optical sections. Scale bar, 2 µm.

To identify the LLPS factors responsible for recruiting G3BP1 to LDs, we first introduced an HCV Core-expression vector into Huh-7.5.1 cells (Fig. 4A and B). Consistent with a previous report (23), HCV Core expressed alone localized around LDs, whereas G3BP1 was not recruited to LDs and remained diffusely distributed in the cytoplasm. The C-terminal hydrophobic region of the HCV Core is known to be important for LD localization (36). To investigate the effect of core LD localization on G3BP1 recruitment, Huh-7.5.1 cells were transfected with either full-length Core (aa1-191) or two types of C-terminally truncated Cores (aa1-111, aa1-123), and the localization of LD, Core, and G3BP1 was examined using immunofluorescence microscopy (Fig. S5A and S5B). As expected, both C-terminally truncated Core mutants completely lost localization on the LD and either diffused into the nucleus or formed amorphous clumps in the cytoplasm. In these cells, G3BP1 was colocalized with Core in the amorphous clumps, and no LD localization was observed. Another plasmid transfection experiment confirmed that expressing only E2, the viral envelope protein, did not cause LD localization of G3BP1 (Fig. S5C).

Both G3BP1 and HCV Core are RNA-binding proteins (12, 37) and have been reported to interact with the viral RNA (37, 38). During HCV infection, viral RNA levels dramatically increased between 36 and 60 hpi (Fig. 4C) (39), which coincided with the timing of G3BP1 recruitment to LDs (Fig. 2A). Based on these observations, we hypothesized that G3BP1 and the HCV Core undergo RNA-dependent phase separation. To test this hypothesis, a viral subgenomic replicon (SGR) RNA containing 5′ and 3′ UTRs of the HCV genome but lacking the coding region of structural proteins was cotransfected into Huh-7.5.1 cells with or without a Core expression vector. Localization of HCV Core and G3BP1 was analyzed by immunofluorescence confocal microscopy (Fig. 4D, E, and F). In cells expressing wild-type SGR RNA (SGR^WT^ RNA), G3BP1 and Core showed prominent colocalization at peri-LD ring structures. At the same time, in some Core-positive cells, although less frequently, LDs without G3BP1 localization were observed, as were stress granules around LDs. In cells where Core was not detected, unlike in Core-positive cells, the localization of G3BP1 around lipid droplets was not prominent (Fig. S6). When an SGR variant in which the translation initiation codon was replaced with a stop codon, and therefore does not express viral protein (SGR^protein-less^ RNA) (Fig. 4D and E), the extent of colocalization of G3BP1 and HCV Core on LDs was comparable (Fig. 4F). PCC values of HCV Core with no SGR RNA, SGR^protein-less^ RNA, and SGR RNA^WT^ were 0.20 ± 0.15, 0.46 ± 0.11, and 0.53 ± 0.08 (mean ± standard deviation, 100 × 100 pixel areas from seven different cells for each group), respectively. Neither single transfection of either SGR RNA nor NanoLuc RNA caused redistribution of G3BP1 (Fig. S7). These results suggest that the recruitment of G3BP1 to peri-LD rings does not require HCV E1, E2, p7, or NS proteins.

Taken together, our data indicate that at the late stage of HCV infection, G3BP1 is recruited to LDs through an LLPS-dependent mechanism, and that the minimal requirements for this recruitment are the presence of HCV Core protein and viral RNA.

## DISCUSSION

In this study, we demonstrated that G3BP1 is recruited to LDs through an LLPS-dependent mechanism in HCV-infected cells, and that the minimal viral components required for this recruitment are the viral Core protein and RNA. We propose that the peri-LD ring represents a biomolecular condensate rather than a conventional protein complex. This concept provides a mechanistic explanation for how viruses can reorganize host factors using only a limited set of viral proteins.

**Fig 5:**
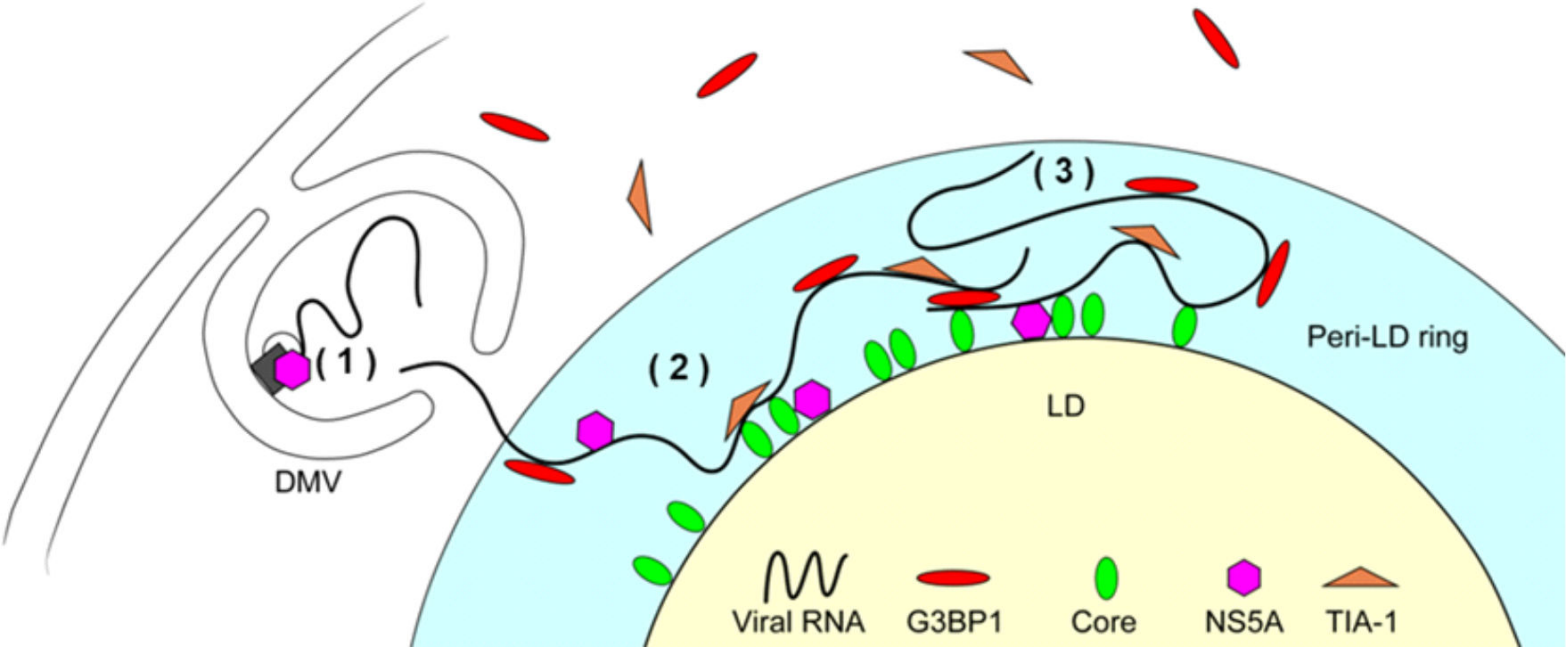
A hypothetical model for the assembly of peri-LD ring structures consisting of HCV Core, the viral RNA, and SG proteins. In HCV-infected cells, Core proteins translated from viral RNA are transported to and accumulate on the surface of LDs (1). Viral NS3–NS5B proteins form replication complexes within cytoplasmic double-membrane vesicles (DMVs), where viral genomic RNA replication occurs. Newly synthesized HCV genomic RNA is released from DMVs together with NS proteins, including NS5A (2). Binding between Core and NS5A leads to the transport of not only NS5A but also the genomic RNA to the vicinity of LDs. This process occurs via a mechanism independent of LLPS (3). The locally increased concentration of long single-stranded HCV genomic RNA around LDs selectively recruits G3BP1 to the LD periphery through LLPS. Other SG proteins, such as TIA-1, are similarly recruited through LLPS mechanisms. Consequently, a thin layer of biomolecular condensates composed of Core, viral RNA, G3BP1, TIA-1, and additional SG-associated proteins is formed around LDs.

A schematic model of peri-LD ring formation is shown in Fig. 5. HCV genomic RNA is synthesized by the replicase complex within double-membrane vesicles that are continuous with the ER membrane and is thought to be delivered to LDs in association with NS5A (40). The localization of Core to LDs is essential for the recruitment of both NS5A and HCV RNA (24). NS5A is retained on LDs by mechanisms independent of LLPS, such as direct binding to Core (40). The long single-stranded HCV RNA likely undergoes phase separation with G3BP1, a central scaffolding protein in SG assembly, which in turn promotes the recruitment of other LLPS-prone host proteins, such as TIA-1, leading to the formation of biomolecular condensates surrounding LDs.

Treatment with 1,6-HD, an inhibitor of LLPS-mediated interactions, completely abolished the localization of SG proteins, G3BP1 and TIA-1, around LD rings, while it did not affect the localization of HCV Core or NS5A. Although cytotoxicity and off-target effects are reported for 1,6-HD (41), its effect on the localization of G3BP1 was reversible (Fig. S4A), suggesting that cellular conditions were restored after the removal of the drug. Additionally, the effect of CHX, or another LLPS inhibitor with a different mechanism of action, on the localization of G3BP1 was similar to that of 1,6-HD. Therefore, it appears that the effect of 1,6-HD observed in this study was unlikely to be due to cell necrosis and/or off-target cytotoxic effects. The viral Core is known to localize on the LD surface by itself (Fig. 4B) (36), whereas NS5A localizes to LDs in a Core-dependent manner (40). These results suggest that the HCV Core acts as a scaffold for the formation of peri-LD rings, which selectively enrich specific proteins and viral RNAs through both classical and LLPS-based interactions. The Core lacking the C-terminal hydrophobic domain failed to localize onto the LDs, forming amorphous clumps in the cytoplasm along with G3BP1 instead (Fig. S5B). As ectopic or overexpression of a protein with intrinsically disordered regions often causes non-specific condensation or aggregation (42, 43), it is not known whether those amorphous clumps are SG-like biomolecular condensates or irreversibly formed aggregations. In any case, this finding in the present study indicates that Core is the major determinant of the localization site of G3BP1 during the HCV life cycle.

Even though there are abundant cellular RNAs in the cytoplasm, they do not seem to be key factors to promote their LLPS in HCV infection. Since the UTRs of HCV genomic RNA have unique secondary structures of RNA, it might be the key to further elucidate the molecular mechanisms of its specificity by comparing the UTRs of HCV and other flaviviruses, such as the Zika virus (16, 18), in which SG assembly is inhibited in the late phase of infection.

The recruitment of SG components to peri-LD rings in HCV-infected cells displays several features unique to the viral life cycle. First, unlike many RNA viruses, HCV infection appears to retain the capacity to assemble SGs. In most RNA virus infections, the accumulation of double-stranded RNA intermediates transiently induces SG formation during the early phase, which is sensed by innate immune receptors. Subsequently, these viruses suppress or dismantle SGs by targeting the cellular stress response—often by cleaving or sequestering G3BP1. Indeed, accumulation of viral RNA typically triggers G3BP1 condensation (44), and several viruses encode proteases that directly cleave G3BP1, including poliovirus (45), coxsackievirus B3 (46), encephalomyocarditis virus (47), feline calicivirus (48), foot-and-mouth disease virus (49–51), and African swine fever virus (52). Porcine epidemic diarrhea virus hijacks caspase-8 to cleave G3BP1 through an unknown mechanism (53). Other viruses, such as Semliki Forest virus and chikungunya virus (54–56), mammalian orthoreovirus (57), murine norovirus (58), SARS-CoV-2 (59, 60), and pseudorabies virus (61), also interact with G3BP1 and alter its localization. In contrast, HCV-infected cells retain the competence to reassemble SGs even at late stages, when virion assembly occurs (Fig. 2) (27, 62). Consistently, arsenite treatment at 48 hpi induced SG formation in nearly all infected cells (Fig. 1) (22), demonstrating that HCV infection preserves SG assembly potential. By contrast, viruses that cleave or sequester G3BP1 generally fail to reassemble SGs under the same conditions.

Second, unlike most biomolecular condensates that exhibit granular morphology, the peri-LD ring forms a thin, continuous layer surrounding the LD surface. A comparable example of SG protein recruitment around membranous structures has been reported in orthoreovirus infection, in which several SG proteins, including G3BP1, relocalize to the periphery of viral replication factories (57). Because this process requires at least two viral proteins, μNS and σNS, the recruitment of G3BP1 in that context likely does not depend on RNA-driven LLPS, as in HCV infection. Nevertheless, because G3BP1 recruited to the surface of orthoreovirus factories can subsequently promote the recruitment of other LLPS-prone host proteins, comparing these regions with the peri-LD rings in HCV infection may yield new mechanistic insights. Given G3BP1’s intrinsic ability to form large SGs, the reason why its recruitment in HCV infection results in only a thin LD-associated layer, rather than large SG aggregates, remains unclear. One possible explanation is that the local RNA concentration near the LD surface surpasses the LLPS threshold for G3BP1 condensation due to the presence of RNA-binding viral proteins, such as Core and NS5A. Alternatively, because SGs are known to consist of a stable core surrounded by a dynamic shell (63), the peri-LD region may correspond to an SG-like shell structure. Since most HCV-infected cells with G3BP1 accumulation around LDs still retain cytoplasmic G3BP1 (Fig. 1B), sequestration alone is unlikely to explain the phenotype. Previous studies have suggested that G3BP1 and TIA-1 are involved in the production of infectious virions at late infection stages (22), raising the possibility that the peri-LD ring contributes to virion assembly.

LLPS typically drives the formation of condensates that selectively concentrate specific molecules while excluding others. It is therefore tempting to speculate that the peri-LD ring acts as a selective microdomain that concentrates components required for genome packaging and virion assembly. This notion is consistent with our observation that the minimal viral factors required for peri-LD ring formation are the viral RNA and Core protein. HCV genomic RNA synthesized by the replication complex is thought to be recruited to LDs through NS5A (5). The accumulation of long single-stranded RNA around LDs likely drives LLPS, forming the peri-LD ring. Supporting this model, we recently identified two RNA-binding host factors, YBX1 and RPL17, which are recruited to LDs and contribute to HCV genome packaging (31). Alternatively, the peri-LD ring may play a role in viral modulation of LD homeostasis. We recently reported that HCV infection decreases LD number but increases their size (64), likely through LD fusion. The peri-LD ring may facilitate this morphological change. Elucidating the roles of the four classes of intracellular structures (Classes I–IV) observed in this study within the viral life cycle is considered important for understanding the regulatory mechanisms of each step in the HCV life cycle. For example, Class IV tends to increase the local concentration of newly generated viral genomic RNA and Core protein, suggesting it is a suitable environment for particle formation.

In conclusion, we identified a novel LLPS-dependent biomolecular condensate that forms around LDs in HCV-infected cells. Its precise composition and functional role remain to be fully elucidated, but our findings suggest that this region represents a specialized LLPS-based domain rather than a conventional static protein complex. Future studies should explore not only the role of G3BP1 but also its homolog G3BP2, whose contribution to viral processes remains less defined. Given the partial functional redundancy of these proteins in SG formation (65), combined knockout analyses will be necessary to uncover their distinct roles. Recent studies indicate that G3BP1 and G3BP2 exhibit non-overlapping functions in condensate formation (66). Understanding these differences may provide deeper insight into how viruses reprogram host condensate dynamics and could inform novel antiviral strategies.

Overall, our study reveals that HCV hijacks host factors through an LLPS-based mechanism, providing new mechanistic insight into the late stages of the HCV life cycle occurring around LDs and offering potential avenues for antiviral intervention.

## MATERIALS AND METHODS

### Cell culture

The hepatocellular carcinoma cell line Huh-7.5.1, a gift from Francis V. Chisari, The Scripps Research Institute (67), was maintained in Dulbecco’s Modified Eagle Medium (DMEM; Sigma Aldrich) supplemented with 100 U/mL penicillin, 100 µg/mL streptomycin, 1% non-essential amino acids, and 10% fetal bovine serum (FBS) at 37°C in a 5% CO₂ incubator. For experiments involving virus infection or DNA/RNA transfection, DMEM supplemented with 1% non-essential amino acids and 10% FBS but without antibiotics was used.

### Virus infection

Recombinant HCV J6/JFH-1 was prepared as described previously (68, 69). Time-course infection experiments were basically performed as follows, unless otherwise indicated. Huh-7.5.1 cells were seeded in 24-well plates at 1 × 10⁵ cells/well. Twenty-four hours later, cells were inoculated with HCV at an MOI of 1. The time of virus addition was defined as 0 hpi. At the same time, uninfected cells cultured under identical conditions were harvested or fixed as 0 hpi samples. At 2 hpi, the inoculum was replaced with fresh DMEM. From 12 to 60 hpi, cells were harvested or fixed every 12 h. Cells at 72 hpi were excluded from sampling due to increased cell death. For all non-time-course experiments, samples collected at 48 hpi were used.

### Plasmids

For HCV Core protein expression, pCAGGS-Core (70) was used. A plasmid for HCV subgenomic replicon mutant (pUC-SGR-JFH-WT) that retains RNA secondary structures but does not express the viral proteins was constructed as follows: the initiator methionine codon of NS3 in pUC-SGR-JFH1-NanoLuc WT (71) was replaced with a stop codon by PCR-based mutagenesis using the following primers: Forward: 5′-GATACCTAGGCTCCCATCACTGCTTATG-3′; Reverse: 5′-GGGAGCCTAGGTATCATCGTGTTTTTCAAAGGAAAAC-3′. PCR products generated using KOD FX neo (TOYOBO) were digested with *Kpn*I and *Cla*I and ligated into the parental plasmid.

### Drug treatment

For SG induction, HCV-infected and uninfected Huh-7.5.1 cells were treated with 0.5 mM sodium arsenite for 45 min at 37°C. Cycloheximide (CHX; FUJIFILM Wako) and 1,6-hexanediol (1,6-HD; Sigma Aldrich) were used as LLPS inhibitors. CHX was prepared as a stock solution at 100 mg/mL in DMSO, and cells were treated with a final concentration of 100 µg/mL for 30 min. 1,6-HD (Sigma Aldrich) was melted at 60°C and diluted with DMEM to prepare 5% (vol/vol) working solution; cells were treated for 5 min at 37°C. After drug treatment, cells were rinsed with PBS and fixed in 4% paraformaldehyde (Electron Microscopy Sciences) for immunofluorescence microscopy.

### RNA synthesis and transfection

Plasmids, pUC-SGR-JFH1-NanoLuc WT, -SGR-protein-null, and -T7-NanoLuc, were linearized with *Xba*I at 37°C overnight and used as templates for *in vitro* transcription. RNAs were synthesized *in vitro* by a MEGAscript T7 kit (Invitrogen) according to the manufacturer’s protocol, treated with DNase I, and purified by TRI reagent (Molecular Research Center). TransIT-mRNA Transfection Reagent (Thermo Fisher Scientific) was used for RNA transfection. For transfection with plasmid DNAs, Lipofectamine LTX with PLUS reagent (Invitrogen) was used. Cells were harvested at 48 hpi for western blotting or immunofluorescence microscopy. RNA concentrations were measured with NanoDrop ND-1000 (Thermo Fisher Scientific), and 1 µg/µL of RNA was transfected in all experiments.

### Immunofluorescence microscopy

Huh-7.5.1 cells were seeded onto φ12-mm round glass coverslips (MATSUNAMI) pre-coated with 0.1 mg/mL poly-D-lysine (FUJIFILM Wako) for 1 h at room temperature (RT). After washing with PBS, cells were fixed with 4% paraformaldehyde in PBS for 30 min at RT, washed three times with PBS, permeabilized with 0.1% saponin (Tokyo Chemical Industry) in PBS for 10 min, and blocked with 5% bovine serum albumin (FUJIFILM Wako) in PBS containing 0.05% sodium azide for 30 min. Cells were incubated with rabbit anti-G3BP1 (Proteintech, 13057-2-AP; 1:1,000), rabbit anti-NS5A (40) (1:500), or rabbit anti-TIA-1 (Proteintech, 12133-2-AP; 1:100), and anti-HCV Core (40) (2H9; 1:1,000) antibodies in blocking buffer for 30 min at RT, washed, and then incubated with fluorescent secondary antibodies. Lipid droplets were stained with Lipi-Blue (Dojindo; 1:200 in PBS) for 30 min at RT. Coverslips were mounted in 90% glycerol/PBS and sealed with nail polish. Images were acquired on a Leica SP8 confocal microscope equipped with HC PL APO CS2 20×/0.75 dry and 63×/1.4 oil objectives using LAS X software. Brightness adjustment was performed for each image for qualitative data, and RAW data were used for quantitative colocalization analysis.

### Western blotting

Western blotting was performed as previously described with modifications (31). Briefly, cell lysates were separated by SDS-PAGE and transferred onto polyvinylidene difluoride membranes. After blocking, membranes were incubated with a primary antibody: rabbit anti-G3BP1 (Proteintech, 13057-2-AP; 1:10,000), mouse anti-HCV Core (2H9; 1:5,000), rabbit anti-NS3 (GTX131276, GeneTex), or mouse anti-GAPDH (Santa Cruz, 6C5; 1:5,000), overnight at 4°C. After washing, membranes were incubated with an HRP-conjugated secondary antibody (Cell Signaling Technology, 7076S or 7075S) for 1 h, washed, and developed using ECL Select (Cytiva). Signals were detected with a FUSION FX imaging system (Vilber).

### RNA isolation and quantitative RT-PCR

To determine HCV RNA copies, total RNAs were isolated from cells with TRI Reagent, followed by isopropanol precipitation. RNA concentrations were measured by the Nanodrop spectrometer (Thermo Fisher Scientific), then diluted to 0.01 µg/µL. Aliquots of RNAs were subjected to qRT-PCR using the CFX Connect Real-Time System and TaqMan Fast Virus 1-Step Master Mix (Applied Biosystems, 4444434). Primers used were as follows: forward: 5′-GAGTGTCGTGCAGCCTCCA-3′; reverse: 5′-CACTCGCAAGCACCCTATCA-3′.

### Microscopic image analysis

Raw confocal images were exported in TIFF format and analyzed with ImageJ or Fiji software. Line profiles were generated using the Plot Profile function along user-defined lines on merged images containing lipid droplet, G3BP1, and HCV Core channels. For PCC analysis, 100 × 100-pixel ROIs (≈81 µm²) containing peri-LD rings were selected from G3BP1 and HCV Core channels. PCC values were calculated using the Coloc2 plugin in Fiji (https://imagej.net/plugins/coloc-2).

## Data Availability

All data generated or analyzed during this study are included in this published article and its supplemental material. Information on the HCV gene used in the expression plasmids is available in GenBank under accession number AB047639.

## SUPPLEMENTAL MATERIAL

Supplemental material jvi.00780-26-s0001.pdf Fig. S1 to S7; supplemental methods.

ASM does not own the copyrights to Supplemental Material that may be linked to, or accessed through, an article. The authors have granted ASM a non-exclusive, world-wide license to publish the Supplemental Material files. Please contact the corresponding author directly for reuse.
