# Supplementary material for: Assembly of lipid droplet-associated ring structures in hepatitis C virus-infected cells via liquid–liquid phase separation (LLPS) and non-LLPS mechanisms
Source: J Virol. 2026 Jun 26;100(7):e00780-26. doi: 10.1128/jvi.00780-26 (PMC13387004; doi:10.1128/jvi.00780-26)
Supplement: Supplemental material — Fig. S1 to S7; supplemental methods. [file jvi.00780-26-s0001.pdf]

Supplementary Information

Assembly of lipid droplet-associated ring structures in hepatitis C virus-infected cells via liquid–liquid phase separation (LLPS) and non-LLPS mechanisms

Mengyu Jiao,[1] Alu Konno,[1#] Xiaowei Wang,[1] Jie Liu,[1] Masahiko Ito,[1] Shinya Satoh,[1] Ryosuke Suzuki,[2] Yasumasa Iwatani,[1] Tetsuro Suzuki,[1,3 ##]

[1]Department of Microbiology and Immunology, Hamamatsu University School of Medicine, Shizuoka, Japan

[2]Department of Virology II, National Institute of Infectious Disease, Japan Institute for Health Security, Tokyo, Japan

[3] Next Generation Creative Education Center for Medicine, Engineering, and Informatics (Nx-CEC), Hamamatsu University School of Medicine, Shizuoka, Japan

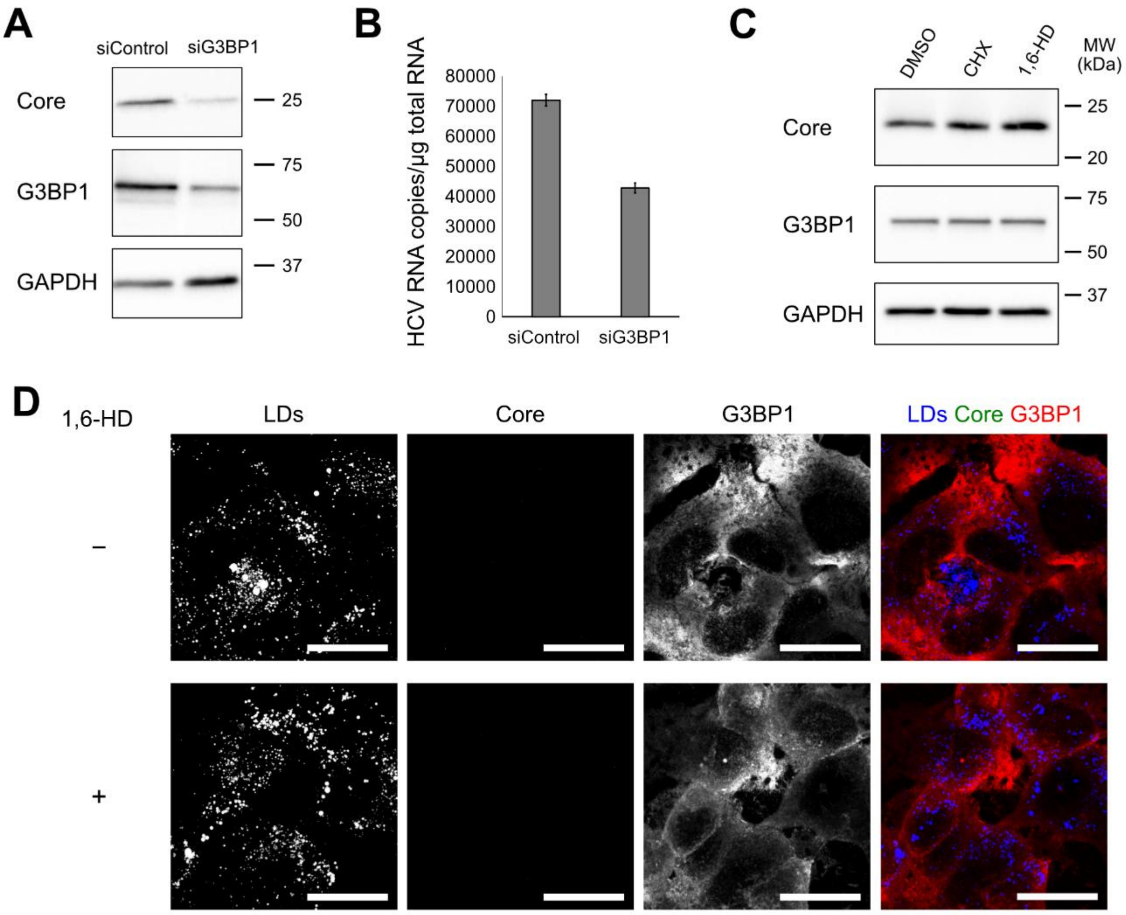
A
siControl
siG3BP1
Core
25
G3BP1
75
50
GAPDH
37
B
HCV RNA copies/μg total RNA
80000
70000
60000
50000
40000
30000
20000
10000
0
siControl
siG3BP1
C
DMSO
CHX
1,6-HD
MW (kDa)
Core
25
20
G3BP1
75
50
GAPDH
37
D
1,6-HD
LDs
Core
G3BP1
LDs Core G3BP1
–
+

Fig. S1. G3BP1 knockdown or treatment with LLPS-modulating drugs in HCV-infected cells. (A, B) siRNA-mediated knockdown of G3BP1. HuH-7 cells persistently infected with HCV JFH-1 (HC-PI cells) were transfected with siRNA against G3BP1 or negative control siRNA. After culturing for 72 h, the culture supernatants collected were inoculated to naive Huh7.5.1 cells, followed by measurement of HCV RNA copies at 48 hpi by quantitative RT-PCR to determine the viral production (B). Expression of Core and G3BP1 was determined by Western blotting (A). (C) Western blotting of Core and G3BP1 proteins in HCV-infected cells treated with LLPS-modulating drugs; 1,6-hexanediol (1,6-HD) and cycloheximide (CHX). (D) Representative confocal images of HCV-infected Huh7.5.1 cells treated with or without 1,6-HD during the early phase of infection (12 hpi). Scale bar, 20 μm.

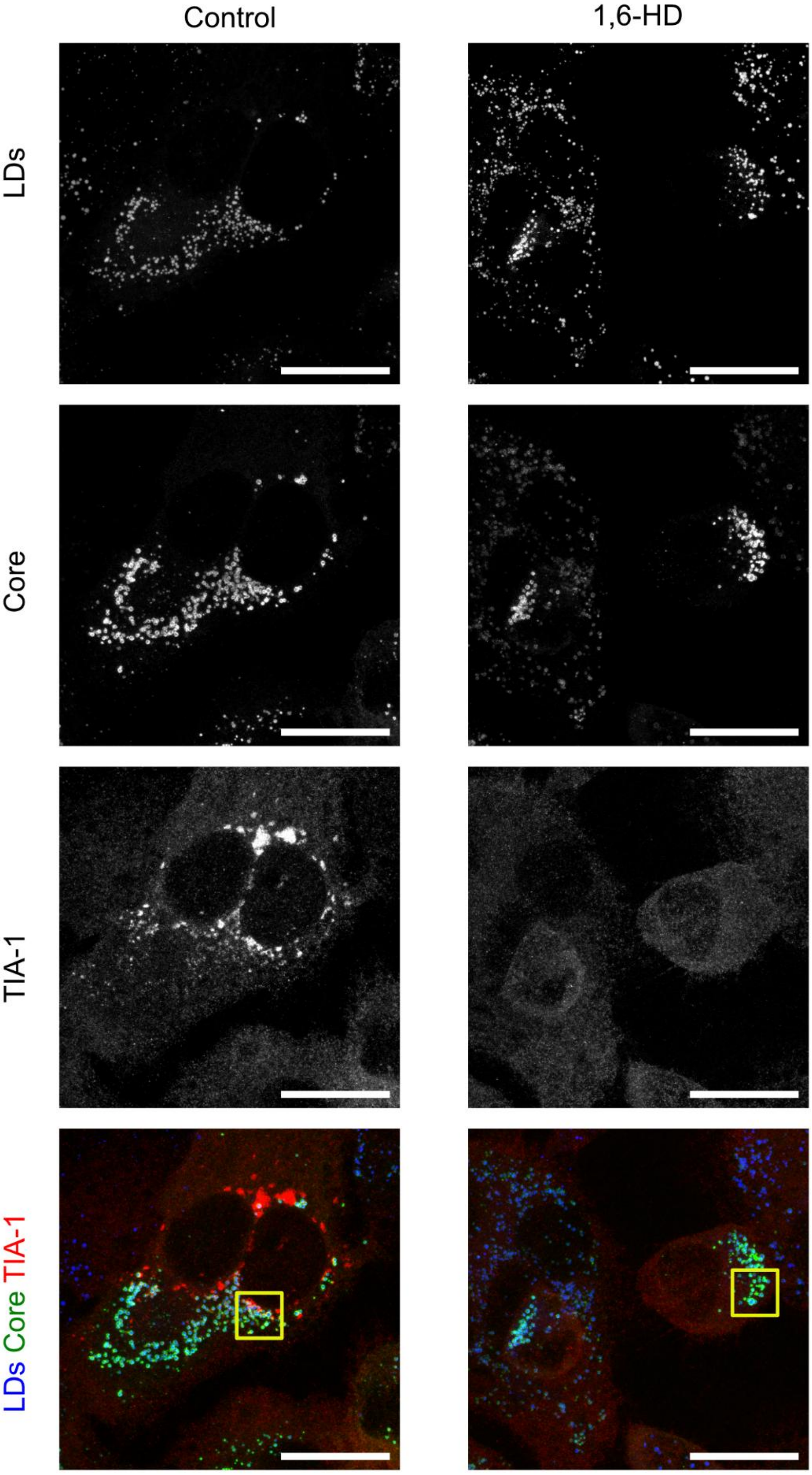

Control
1,6-HD
LDs
Core
TIA-1
LDs Core TIA-1

Fig. S2. Low-magnification confocal images of Core and TIA-1 immunostaining in cells with or without 1,6-hexanediol (1,6-HD). Immunofluorescence confocal images of TIA-1 and G3BP1 in HCV-infected cells with or without 1,6-HD treatment. Lipid droplets (LDs) were labeled with Lipi-Blue. Insets in the merged images correspond to enlarged regions shown in Fig. 3B. LDs: lipid droplets. Scale bar, 20 μm. All experiments were performed twice independently and comparable results were obtained.

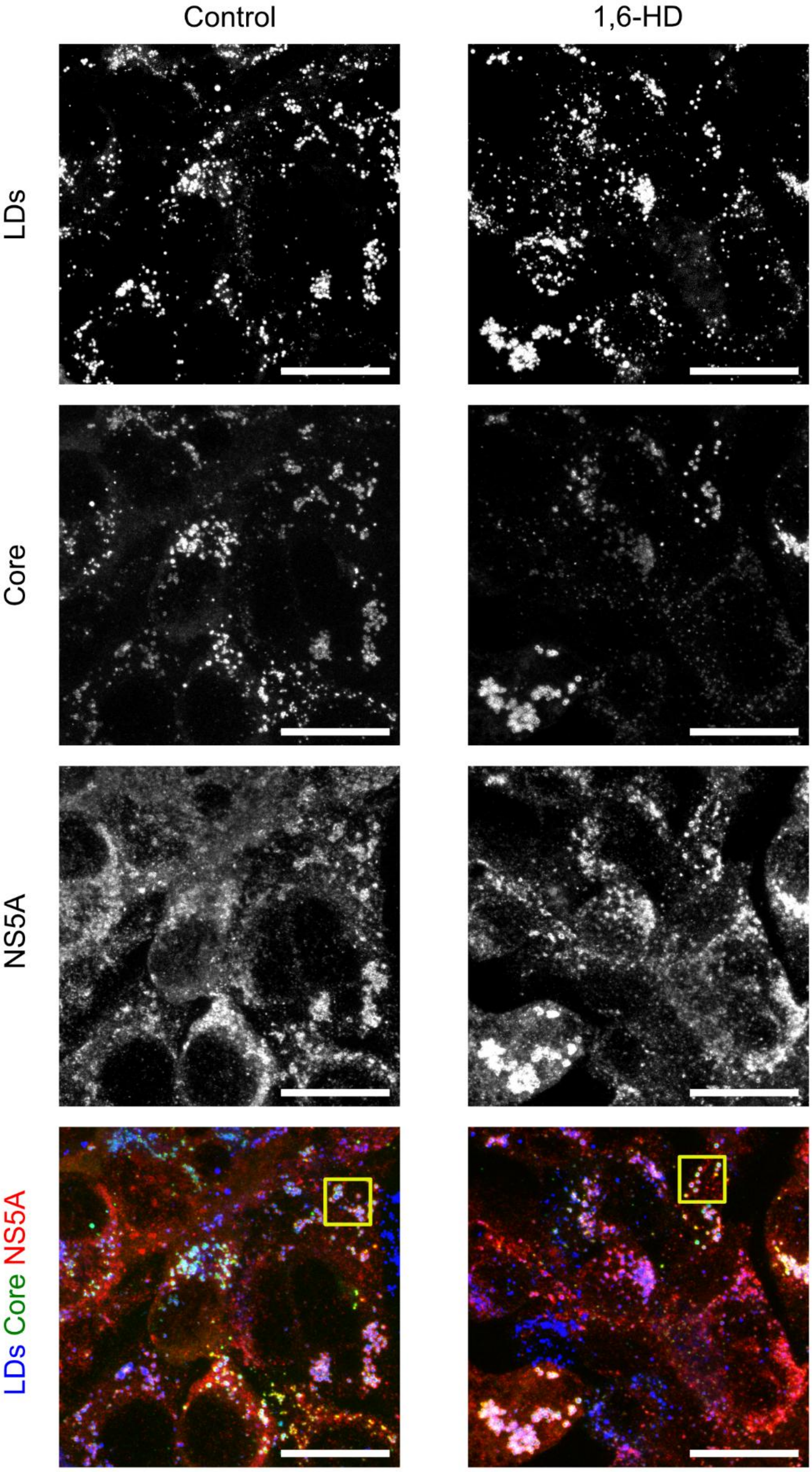
Control
1,6-HD
LDs
Core
NS5A
LDs Core NS5A

Fig. S3. Low-magnification confocal images of NS5A immunostaining in cells with or without 1,6-hexanediol (1,6-HD). Immunofluorescence confocal images of NS5A and G3BP1 in HCV-infected cells with or without 1,6-HD treatment. Lipid droplets (LDs) were labeled with Lipi-Blue. Insets in the merged images correspond to enlarged regions shown in Fig. 3C. LDs: lipid droplets. Scale bar, 20 μm. All experiments were performed twice independently and comparable results were obtained.

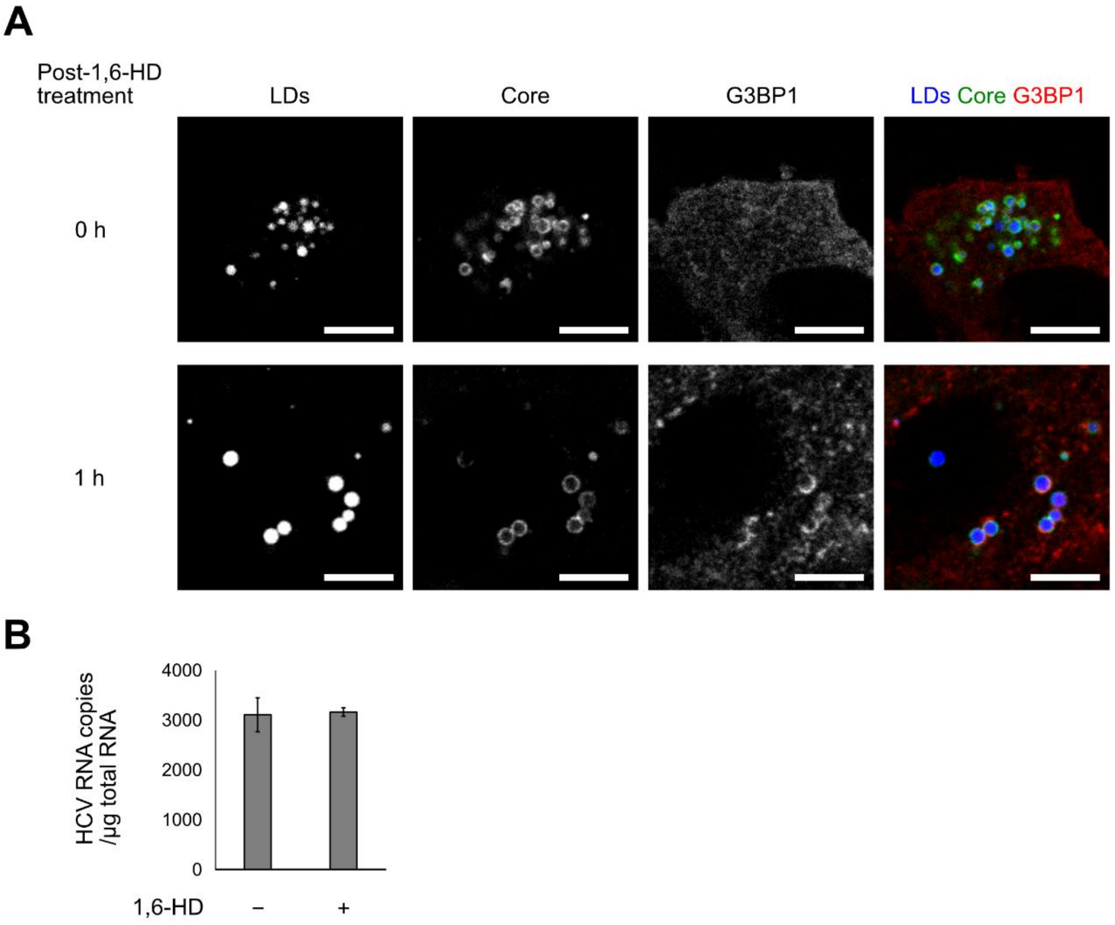
A
Post-1,6-HD treatment
LDs
Core
G3BP1
LDs Core G3BP1
0 h
1 h
B
HCV RNA copies /µg total RNA
4000
3000
2000
1000
0
1,6-HD
–
+

Fig. S4. Long-term effect of transient 1,6-hexanediol (1,6-HD) treatment. (A) Comparison between cells analyzed immediately after 5 min treatment with 1% 1,6-HD (upper panels) versus cells cultured in drug-free medium for 1 h after drug removal (lower panels). Scale bar, 5 μm. (B) Effect of 1,6-HD treatment on HCV production. HC-PI cells were treated with 5% 1,6-HD for 5 min. After removal of the drug, cells were cultured for an additional 48 h and intracellular HCV RNA levels were quantified.

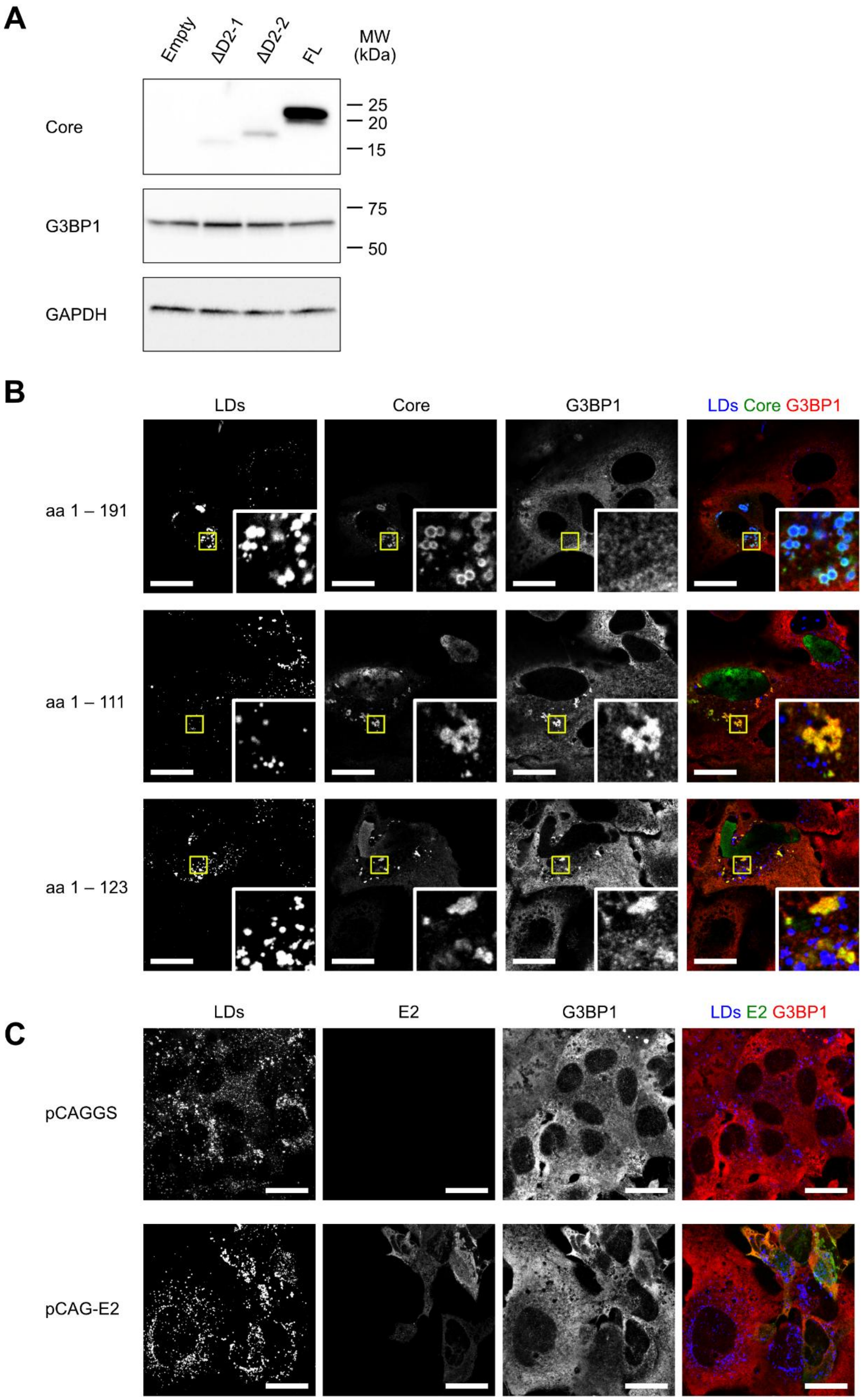

A
Empty
ΔD2-1
ΔD2-2
FL
MW (kDa)
Core
25
20
15
G3BP1
75
50
GAPDH
B
LDs
Core
G3BP1
LDs Core G3BP1
aa 1 – 191
aa 1 – 111
aa 1 – 123
C
LDs
E2
G3BP1
LDs E2 G3BP1
pCAGGS
pCAG-E2

Fig. S5. Effects of truncation mutants of HCV Core or E2 expression on the intracellular localization of G3BP1. (A, B) Detection of HCV Core and G3BP1 by Western blotting (A) and immunofluorescence staining (B) in cells expressing full-length HCV Core (aa 1–191) or C-terminal truncation mutants (aa 1–111 and aa 1–123). (B) Lipid droplets were stained with LipiBlue. Insets in the upper right corner of each panel show enlarged views of the regions outlined by yellow boxes. A single confocal optical section is shown. Scale bars = 20 μm. (C) Representative confocal images of cells expressing HCV E2 protein. Scale bars = 20 μm. All experiments were independently performed twice and comparable results were obtained.

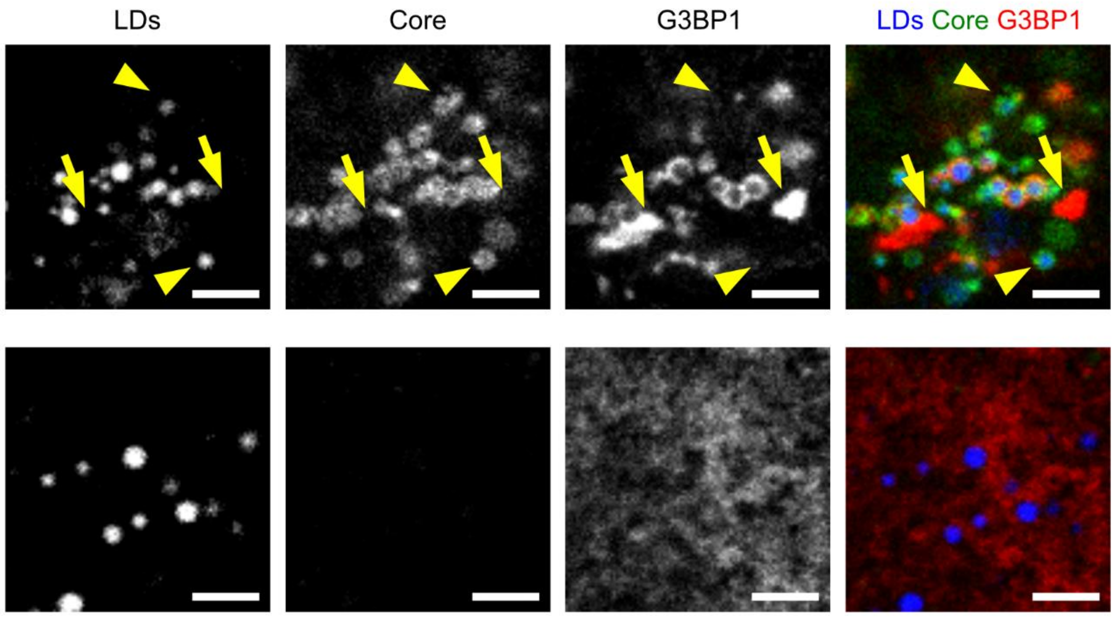

LDs
Core
G3BP1
LDs Core G3BP1

Fig. S6. Intracellular localization of Core and G3BP1 in cells cotransfected with the Core expression plasmid and SGR^{WT} RNA. In Core-positive cells, lipid droplets (LDs) surrounded by colocalized Core and G3BP1 were typically observed, and representative images are shown in Fig. 4F. In addition, although less frequently observed, some Core-positive cells exhibited LDs lacking G3BP1 localization (arrowheads) or stress granules localized adjacent to LDs (arrows) (upper panels). In contrast, in cells in which Core expression was not detected (lower panels), G3BP1 accumulation around LDs was not prominent compared with Core-positive cells. Single confocal optical sections are shown. Scale bar = 2 μm.

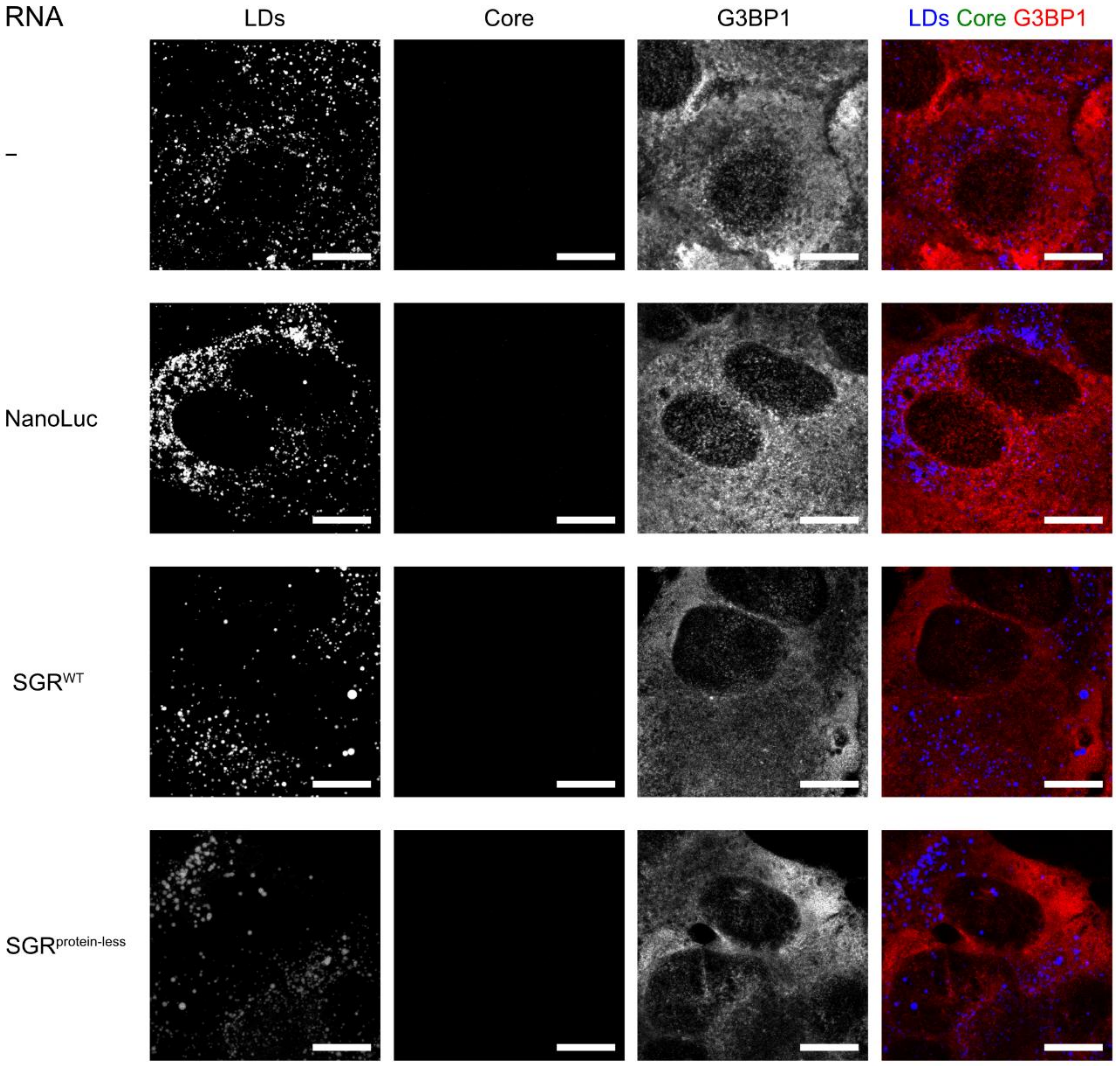

RNA
LDs
Core
G3BP1
LDs Core G3BP1
–
NanoLuc
SGR^{WT}
SGR$^{protein-less}$

Fig. S7. Effects of transfection with SGRWT RNA, SGR protein-deficient RNA, or NanoLuc RNA alone on G3BP1 localization. Huh7.5.1 cells were transfected with NanoLuc mRNA, SGRWT RNA, or SGR protein-deficient RNA, and immunofluorescence confocal images of G3BP1 were obtained at 48 h post-transfection. Lipid droplets (LDs) were stained with Lipi-Blue. Scale bars = 10 μm. All experiments were independently performed twice with comparable results.

Supplementary Materials and Methods

Plasmids

The expression plasmids for the full-length HCV Core (199 aa; C191) with an N-terminal HA tag, and the C-terminal Core deletion variants, C111 (aa 1-111) and C123 (aa 1–123), were constructed by replacing the DHFR-HA-Ub gene region of pUPRC191, pUPRC123 and pUPRC111 (2) with the HA-spacer (MYPYDVPDYGGGGS). The resultant plasmids were named pCAG-HAC191, pCAG-HAC123 and pCAG-HAC111, respectively. The expression plasmid for HCV E2 (pCAG-JFH1-E2) was constructed based on pCAGC-NS2/JFH1 (Supplementary ref.).

Expression of HCV proteins

Huh-7.5.1 cells were seeded at a density of 1×10^5 cells per well in 24-well plates and cultured for 24 h, The cells were then transfected with 0.5 μg of expression plasmid for HCV Core or E2 or the empty vector pCAGGS using the Lipofectamine LTX with PLUS reagent (Invitrogen) according to the manufacturer's instructions. Forty-eight hours after transfection, cells were harvested for Western blotting or immunofluorescence microscopy. A mouse anti-E2 antibody (SLT-8) (4) was used for E2 detection.

siRNA-mediated knockdown

HCV-infected Huh7 cell were seeded at a density of 3×10^5 cells per well in 12-well plates and cultured for 24 h. The cells were then transfected with siG3BP1 (Thermo Fisher Scientific, 5′-GUCUGAAUGUCGAAGAGAAtt-3′), or with a non-targeting control siRNA using the Lipofectamine RNAiMAX (Thermo Fisher Scientific) according to the manufacturer's instructions. Seventy-two h after transfection, the cells were harvested for western blotting. The culture supernatant was collected to inoculate naïve Huh 7.5.1 cells. Forty-eight h after inoculation, intracellular HCV RNA copy numbers in the naïve cells were determined by quantitative RT-PCR.

Supplementary Reference

Suzuki R, Saito K, Kato T, Shirakura M, Akazawa D, Ishii K, Aizaki H, Kanegae Y, Matsuura Y, Saito I, et al. 2012 Trans-complemented hepatitis C virus particles as a versatile tool for study of virus assembly and infection. Virology 432:29–38. https://doi.org/10.1016/j.virol.2012.05.033
